# Distribution and Afferent Effects of Transplanted mESCs on Cochlea in Acute and Chronic Neural Hearing Loss Models

**DOI:** 10.1155/2021/4956404

**Published:** 2021-06-21

**Authors:** So-Young Chang, Hee-Won Jeong, Eunjeong Kim, Jae Yun Jung, Min Young Lee

**Affiliations:** ^1^Beckman Laser Institute Korea, Dankook University, Cheonan 31116, Republic of Korea; ^2^Medical Laser Research Center, Dankook University, Cheonan 31116, Republic of Korea; ^3^Department of Biological Science, College of Science & Technology, Dankook University, Cheonan 31116, Republic of Korea; ^4^Department of Otolaryngology-Head & Neck Surgery, College of Medicine, Dankook University, Cheonan 31116, Republic of Korea

## Abstract

Hearing loss is a sensory deprivation that can affect the quality of life. Currently, only rehabilitation devices such as hearing aids and cochlear implants are used, without a definitive cure. However, in chronic hearing-deprived patients, in whom secondary auditory neural degeneration is expected, a relatively poor rehabilitation prognosis is projected. Stem cell therapy for cochlear neural structures would be an easier and feasible strategy compared with cochlear sensory cells. Considering the highly developed cochlear implantation technology, improving cochlear neural health has significant medical and social effects. Stem cell delivery to Rosenthal's canal in an acutely damaged mouse model has been performed and showed cell survival and the possibility of differentiation. The results of stem cell transplantation in chronic auditory neural hearing loss should be evaluated because neural stem cell replacement therapy for chronic (long-term) sensorineural hearing loss is a major target in clinics. In the present study, we established a mouse model that mimicked chronic auditory neural hearing loss (secondary degeneration of auditory neurons after loss of sensory input). Then, mouse embryonic stem cells (mESCs) were transplanted into the scala tympani and survival and distribution of transplanted cells were compared between the acute and chronic auditory neural hearing loss models induced by ouabain or kanamycin (KM), respectively. The mESC survival was similar to the acute model, and perilymphatic distribution of cell aggregates was more predominant in the chronic model. Lastly, the effects of mESC transplantation on neural signal transduction observed in the cochlear nucleus (CN) were compared and a statistical increase was observed in the chronic model compared with other models. These results indicated that after transplantation, mESCs survived in the cochlea and increased the neural signaling toward the central auditory pathway, even in the chronic (secondary) hearing loss mouse model.

## 1. Introduction

Hearing loss is a sensory deprivation that can affect the quality of life. Hearing loss has significant social implications, and the association of hearing loss with cognitive dysfunction has been recently introduced [1]. Therapeutic approaches to cure chronic sensorineural hearing loss without a rehabilitation device do not currently exist due to the lack of regenerative function of sensory hair cells in the cochlea. Therefore, rehabilitation devices, such as hearing aids and cochlear implants, are used to reduce the degree of hearing loss and increase sound perceptions [2].

Sound transduction is a complicated process performed by sensory and neural structures. Therefore, sensory and neural structures are well organized to properly transduce sound. When bypassing the sensory part among these structures, for example, using hearing aids or cochlear implants to augment hearing, maintaining the health of neural hearing structures remains important [3]. Therefore, in clinics, early rehabilitation is recommended because neural structures gradually degenerate due to plasticity and rearrangement of the auditory neural pathway occurs after sensory deprivation. Conversely, in chronic hearing-deprived patients, in whom secondary auditory neural degeneration is expected, a relatively poor rehabilitation prognosis is projected.

Stem cells, by definition, are cells that can proliferate and differentiate [4]. Pluripotent stem cells can differentiate into various lineages and are an expected source for many different intractable diseases [4]. Trials to generate cochlear sensory hair cells from pluripotent stem cells are ongoing. In several studies, successful differentiation of hair cell-like cells using direct *in vitro* differentiation or organoids has been performed [5–8]. However, significant obstacles to stem cell transplantation and differentiation for sensory cell replacement therapy exist such as a hostile environment and limited survival time window (insufficient time to differentiate into target cells) [9]. The differentiation techniques for a specific auditory neuron from pluripotent stem cells have previously been described [10–13]. In contrast to sensory cells, which are located in scala media (fluid cavity with high potassium concentration), neural structures are located in an environment relatively favorable for stem cells. In addition, scala tympani, which could be a route for stem cell delivery to neural structures such as Rosenthal's canal, is filled with fluid similar to that of cellular ionic concentration. Therefore, stem cell therapy for cochlear neural structures would be an easier and feasible strategy compared with cochlear sensory cells. Considering the highly developed cochlear implantation technology, improving cochlear neural health would significantly expand the indication for and efficiency of hearing rehabilitation using cochlear implants.

Stem cell delivery to Rosenthal's canal has previously been attempted. Stem cell survival in both Rosenthal's canal and perilymphatic space and the possibility of differentiation have been proposed and performed in acutely damaged mouse models [14]. However, in clinics, the major target for stem cell replacement therapy for hearing rehabilitation is chronic (long-term) sensorineural hearing loss. Auditory nerve degeneration can be divided into acute and chronic processes, and studies using acute and chronic nerve injury mouse models for restoring or rescuing the hearing function have been reported. In several published papers, type 1 SGN reportedly is the damaged auditory neuron after acute [15, 16] and chronic [17] nerve injuries. Acute auditory neural damage and chronic auditory neural damage are completely different conditions for transplanted cells. In the chronic model, damaged or lost cells are replaced by other cells and scar formation is completed, which limits the distribution and function of transplanted cells. Thus, the outcome of stem cell transplantation in chronic auditory neural hearing loss should be evaluated.

In the present study, a mouse model was established that mimicked chronic auditory neural hearing loss (secondary degeneration of auditory neurons after loss of sensory input). Then, mouse embryonic stem cells (mESCs) were transplanted into the scala tympani and survival and distribution of transplanted cells evaluated and compared to the acute auditory neural hearing loss model induced by ouabain. Lastly, the effects of mESC transplantation on neural signal transduction, observed in the cochlear nucleus (CN), were evaluated.

## 2. Materials and Methods

### 2.1. Animals

**Figure 1:**
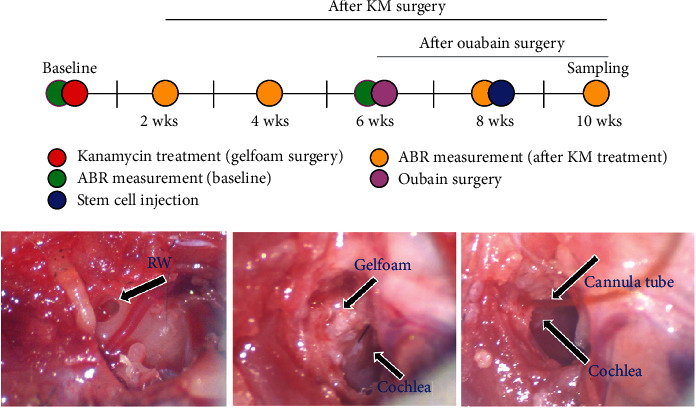


Twenty-one male, 5-week-old, adult C57BL/c6 mice were used in this study. The animals were divided into five groups: no treatment control (*n* = 4), kanamycin (KM) only (*n* = 5), KM plus stem cell (SC) (*n* = 6), ouabain only (*n* = 3), and ouabain plus SC (*n* = 3). In the ototoxic treatment groups, ototoxic agents were delivered only to their left ear (unilateral). Before the surgery to induce secondary auditory nerve degeneration after ototoxic damage and mESC transplantation, the mice were anesthetized with an intramuscular injection of a mixture solution (0.1 mL/20 g) prepared by diluting a 1 : 3 mixture of normal saline from 3 : 1 mixture of Zoletil (Virbac, Carros Cedex, France) and Rompun (Bayer, Leverkusen, Germany). All experimental surgeries including mESC implantation were performed in the left ear only. Hearing was measured using the auditory brainstem response (ABR) and assessed before and after ototoxic agent injection and mESC transplantation as shown in Figure 1. Animal care and all experiments were performed according to the required application protocol and approved by Dankook University Animal Care and Use Committee (IACUC-DKU).

### 2.2. Stem Cells and Stem Cell Culture

The ESCs tagged with green fluorescent protein (GFP), donated by Prof. Hosup Shim (Dankook University, Cheonan, Korea), were used in this study. The same culture method for maintenance followed the previously published method due to the same donated cells [18]. Cells were cultured in gelatin-coated plates without feeder cells and maintained in ESC medium consisting of high-glucose Dulbecco's modified Eagle's medium (DMEM) (Sigma-Aldrich, St. Louis, MO, USA) supplemented with 15% (*v*/*v*) heat-inactivated fetal bovine serum (FBS) (ATCC, Manassas, VA, USA), 0.1 mM *β*-mercaptoethanol (Gibco, Invitrogen, Carlsbad, CA, USA), 0.1 mM GlutaMAX (Gibco), 0.1 mM ES qualified nonessential amino acid (NEAA) (Welgene, Daegu, Korea), 1% penicillin-streptomycin (PS) (ATCC), 1000 U/mL leukemia inhibitory factor (Merck Millipore, Burlington, MA, USA), 0.033% CHIR99021 (Tocris Bioscience, Bristol, UK), and 0.125% PD035901 (Tocris Bioscience) at 37°C in a 5% CO_2_ humidified incubator.

### 2.3. Auditory Neural Hearing Loss Mouse Model (Acute and Chronic)

Two auditory neural hearing loss mouse models were created. First, an acute model was designed to mimic acute neural hearing loss with intact sensory structures. Second, a chronic model was created to resemble the chronic status of sensorineural hearing with secondary neural degeneration resulting from the lack of sensory input from the cochlea. Ouabain (Sigma-Aldrich, St. Louis, MO, USA) and KM (Sigma-Aldrich, St. Louis, MO, USA) were used to develop the acute and chronic mouse models, respectively. To induce auditory neurodegeneration in the mouse, the posterior wall of the left ear was dissected until the bulla appeared. Next, the bulla was carefully removed until the round window of the cochlea was exposed. A Gelfoam soaked with ouabain (5 *μ*L of a 1 mM solution) or KM (5 *μ*L of a 150 mg/kg solution) was placed into the round window to deliver the drug through the scala tympani via a microcannula tube connected to a Hamilton syringe. After the surgery was completed, the incision was sutured and the mice stabilized on a warm pad until normal movement. To evaluate the hearing changes, the ABR was measured before and 2 weeks after ouabain treatment. After KM treatment, the ABR was measured before treatment and at 2, 4, and 8 weeks after treatment.

### 2.4. Green Fluorescent Protein- (GFP-) Tagged mESC Transplantation

For the mESC transplantation experiment, at 2 weeks and 8 weeks after auditory nerve degeneration induction in the acute and chronic mouse models, respectively, green fluorescent protein- (GFP-) tagged mESCs were transplanted into both models. The surgical technique followed a previously published report in a way that our research team continued it [18]. In brief, the mice were placed on the lateral left side after anesthesia. The posterior wall of the left ear was cut again and the round window was identified. Next, a total volume of 3 *μ*L of mESCs (2 × 10^4^ cells/*μ*L) was injected into the scala tympani of the cochlea at 8 weeks after KM treatment. After transplantation, the incision was sutured in the same manner as in the method for inducing auditory nerve degeneration.

### 2.5. ABR Recordings

To measure hearing changes, the ABR was performed with tone bursts and a specific stimulated frequency (RZ6 Multi-I/O Processor; Tucker Davis Technologies, Alachua, FL, USA). The treated mice were placed in a sound chamber, and three needle electrodes were inserted at the vertex (active) and under both auricles (reference and ground). To evaluate the hearing threshold, tone burst stimulations of 8, 16, and 32 kHz were delivered through a tube inserted into the left ear of the mouse. The ABR waveform was measured by averaging 512 signals for 10 ms/step measured from 80 dB SPL to 10 dB at 5 or 10 dB intervals.

### 2.6. Histological Preparation and Epifluorescence Analysis

Mice were subjected to cardiac perfusion under anesthesia to obtain the cochlea and brain. The cochlea and brain were harvested from mice in each group. The cochleae were postfixed in 4% PFA and then decalcified in 0.1 M EDTA solution for 4 days at room temperature (RT). The cochleae were washed with PBS for 3 h to stop the decalcification and embedded in a paraffin block. The blocks were sliced into 5 *μ*m thick sections and stained with hematoxylin and eosin (H&E). Then, the sections were observed under a microscope (BX53, Olympus, Tokyo, Japan) to evaluate the histological changes in peripheral auditory organs after mESC transplantation in the auditory neural hearing loss model.

The harvested brains were postfixed in 4% PFA overnight at 4°C, and crystallization was prevented by soaking in 10, 20, and 30% (*w*/*v*) serial sucrose solutions until the tissue relaxed. Then, the brains were embedded in the OCT compound (Tissue-Tek, Torrance, CA, USA) and sliced into 5 *μ*m thick sections under cold conditions using a microtome (Leica CM 1860, Wetzlar, Germany). The prepared slides were stained for epifluorescence imaging.

Briefly, after drying the slides for 10 min, the slides were washed three times with PBS for 10 min and blocked for 1 h at RT with 5% normal goat serum (NGS) (Vector Laboratories, Burlington, Canada) and 0.3% Triton X-100 (Sigma-Aldrich) to prevent nonspecific binding. To identify GFP-positive cells inside the cochlea, the slides were incubated with neurofilament antibody (1 : 200; Merck, Darmstadt, Germany) overnight at 4°C without GFP. The following day, the slides were washed three times with PBS for 5 min and then incubated with secondary antibody (1 : 1000; Alexa Fluor 568-conjugated goat anti-chicken IgY; Invitrogen, Waltham, MA, USA) for 1 h. Nuclei were stained using 4′,6-diamidino-2-phenylindole (DAPI).

To evaluate vesicular glutamate transporter 1 (VGLUT1) expression in CN after mESC implantation in the auditory neural hearing loss model, samples were incubated with VGLUT1 antibody (1 : 200; Synaptic Systems, Göttingen, Germany) overnight at 4°C. The next day, samples were washed as described above. Animals without any treatment were sacrificed as controls. Then, the samples were incubated with secondary antibody (1 : 1000; Alexa Fluor 568-conjugated goat anti-Rabbit IgG; Invitrogen) for 1 h and visualized using DAPI. A confocal microscope (Olympus FW3000, Tokyo Japan) was used to obtain representative images. In the acquired images, the intensity of VGLUT1 within the CN was measured using ImageJ software 1.43u (National Institutes of Health, Bethesda, MD, USA).

## 3. Statistical Analysis

The results were expressed as means ± standard deviation (SD) and analyzed using GraphPad Prism software (La Jolla, CA, USA) or SPSS software (IBM; Armonk, NY, USA). The Kolmogorov-Smirnov test was used to determine whether data were parametric. Two-way ANOVA was performed as well as Bonferroni tests as posttests to analyze the ABR thresholds. Significant differences between the ototoxin-treated group and ESC-transplanted groups were statistically analyzed using two-tailed paired *t*-tests. To analyze the VGLUT1 expression, the Kruskal-Wallis test, a nonparametric one-way ANOVA, was performed and Dunn's test for multiple comparisons was used as the posttest. A *p* value < 0.05 was considered statistically significant.

## 4. Results

### 4.1. ABR Threshold Change after mESC Transplantation in Both Auditory Neural Hearing Loss Models

**Figure 2:**
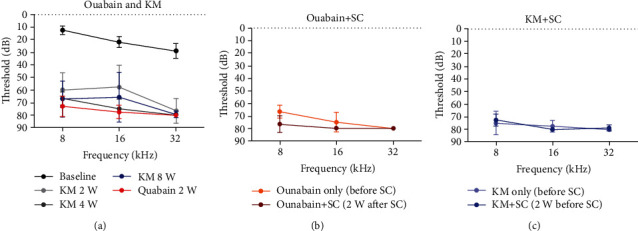


After ouabain (acute model) and KM (chronic model) injection, the ABR threshold in the ipsilateral ear increased significantly at all frequencies (8, 16, and 32 kHz) (Figure 2(a)).

In the acute neural hearing loss model, mESC was transplanted at 2 weeks after ouabain injection. In mice treated with ouabain and mESCs, hearing improvement was not observed compared with control (ouabain only; prior to mESC transplantation) (two-tailed paired *t*-test: 8 kHz: *t* = 3.464, df = 2, *p* = 0.0742; 16 kHz: *t* = 1.000, df = 2, *p* = 0.4226; and 32 kHz: *t* = 1.000, df = 2, *p* = 0.4226) (Figure 2(b)).

After the KM treatment, the degree of hearing loss was variable. The efficiency percentages of successful ototoxic treatment (if >60 dB thresholds were successful hearing loss) rates of 8 kHz and 16 kHz were 78.6% and 92.8% for 32 kHz, respectively. The increased threshold remained unchanged until 8 weeks (two-way ANOVA, Bonferroni post-tests: baseline vs. 8 weeks; 8 kHz: *t* = 13.34, *p* < 0.001; 16 kHz: *t* = 12.29, *p* < 0.001; and 32 kHz: *t* = 11.26, *p* < 0.001), indicating the possibility of chronic hearing deprivation. At 2 weeks after GFP-mESC transplantation into the cochlea through the round window in the chronic auditory neural hearing loss mouse model (8 weeks after KM treatment), ABR was measured to evaluate hearing improvement. However, compared with the chronic auditory neural hearing loss model, improvement in hearing threshold was not observed (two-tailed paired *t*-test: 8 kHz: *t* = 0.5222, df = 3, *p* = 0.6376; 16 kHz: *t* = 1.000, df = 3, *p* = 0.3910; and 32 kHz: *t* = 1.000, df = 3, *p* = 0.3910) (Figure 2(c)). These results showed that mESCs transplanted via the scala tympani did not produce any hearing improvement in the acute or chronic hearing loss model.

### 4.2. Morphological Changes in Peripheral Auditory Organs with or without mESC Transplantation in a Neural Hearing Loss Model

**Figure 3:**
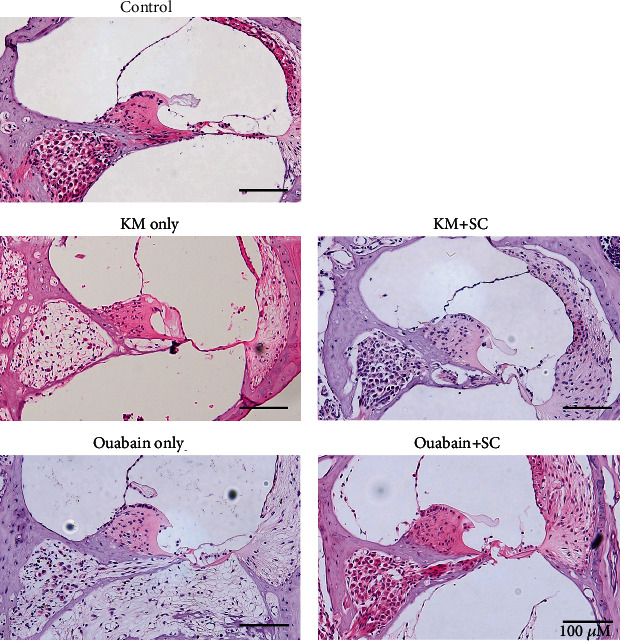


In the acute or chronic auditory neural hearing loss model, the histological changes in the peripheral auditory organs were observed using H&E staining as shown in Figure 3. In KM-treated mice, the organ of Corti showed variable morphologies from a condition in which still cells were present (probably supporting cells) to flat epithelium, compared with the control. In the ouabain-treated mice with acute auditory nerve damage, the organs of Corti showed identical morphologies in which still cells were present. Regarding the morphology of Rosenthal's canal, in both mouse models, a substantial decrease of the spiral ganglion neuron (SGN) density (expanded in all turns) was observed in the majority of animals (2/3 in the acute model and 3/5 in the chronic model; Table 1).

**Figure 4:**
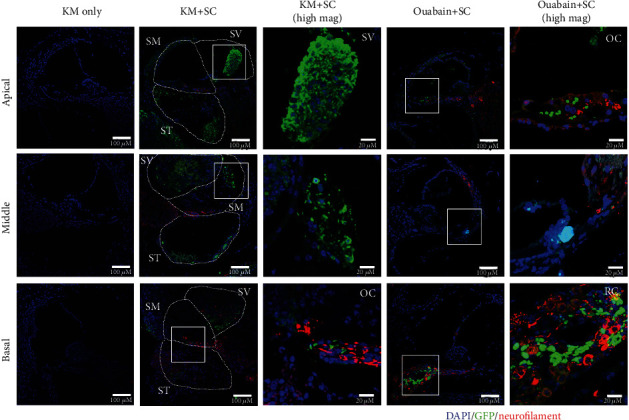


However, after mESC transplantation, cells (probably supporting cells or perhaps transplanted mESC) in the organ of Corti were observed in both acute (ouabain) and chronic (KM) neural hearing loss models (Figure 4). In Rosenthal's canal, half of the mice in the chronic model showed a partial decrease of SGN density (limited to the basal turn) and the other mice (including all mice in the acute model) showed no decrease of SGN density in mESC-transplanted animals (Table 1). These results showed slightly less insulted anatomical structures in mESC-transplanted animals compared with animals without mESC transplantation.

### 4.3. Epifluorescence Analysis of GFP-Tagged mESCs Transplanted into the Cochlea of Mice with Auditory Neural Hearing Loss

To track the delivery pattern and survival of mESCs in the cochlea easily, mESCs tagged with GFP were transplanted into mice with both auditory neural hearing loss models. The GFP expression in peripheral auditory organs was observed in half of the mice in the chronic model and in all mice in the acute model at 2 weeks after mESC transplantation into the scala tympani through the round window. In both neural hearing loss models, mESCs were observed in all turns of cochlea. mESCs were observed in Rosenthal's canal in both neural hearing loss models. Obvious GFP expression was observed in mice in the acute model and weak GFP expression in mice in the chronic model. Limited GFP and neurofilament coexpression was observed, indicating relatively low differentiation of mESCs into neural structures. In mice in the chronic model, especially in the perilymph, transplanted mESCs formed cellular aggregates showing GFP-positive oval structures with varying densities. The structures extended into the scala vestibuli, which is anatomically distant from the initial transplantation site. In mice in the acute mouse model, several mESCs migrated into the organ of Corti and resided adjacent to supporting cells and tunnel of Corti (Figure 4). Based on the shape of the nuclei in the double-stained cells, it seems that not only (some) cell differentiation but also cell fusion may have occurred. Detailed distribution of GFP-positive mESCs is shown in Table 2. Survival of mESCs was adequate in mice in the acute model, and perilymphatic distribution of cell aggregates was more predominant in mice in the chronic model. These results demonstrated successful survival and migration of transplanted mESCs into specific subareas of cochlea in both acute and chronic mouse models of neural hearing loss. Small numbers of cells successfully migrated into the neural structures, which were predicted to be damaged by the two different insults, indicating the possibility of using cell therapy for degenerated neural structures.

### 4.4. Changes of VGLUT1 Expression in CN after mESC Transplantation in Both Auditory Neural Hearing Loss Models

**Figure 5:**
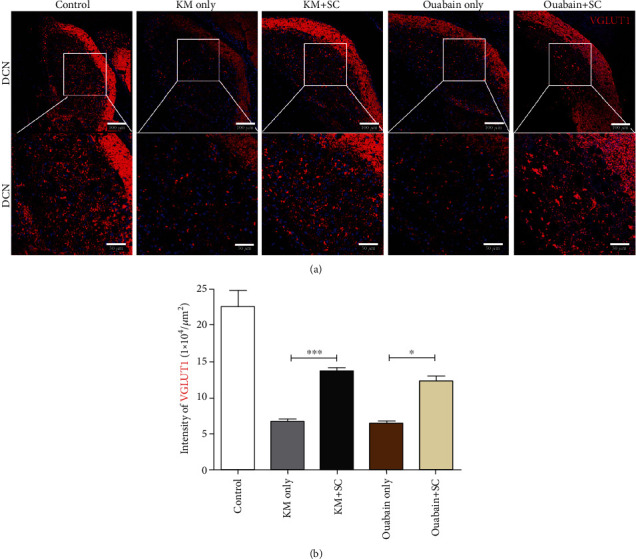


The connection between SGN and CN, which is the proximal central neural structure, was evaluated, and alteration of this connection due to mESC transplantation in both auditory neural hearing loss models was confirmed. The VGLUT1 expression in the dorsal cochlear nucleus (DCN) of the CN within the brainstem, the first structure to the central auditory pathway, was observed (Figure 5). The VGLUT1 expression was decreased in DCN after KM treatment compared with control. However, VGLUT1 expression was increased in animals with mESC transplantation after both KM and ouabain treatments compared with the KM only-treated mice (Figure 5(a)). Based on quantitative analysis, VGLUT1 intensity was statistically greater in mice with mESC transplantation than without mESC transplantation in the chronic auditory neural hearing loss model induced by KM and in mice with mESC transplantation in the acute neural hearing loss model induced by ouabain (one way ANOVA, Kruskal-Wallis statistic: 32.96; Dunn's test for multiple comparisons KM vs. KM plus mESCs, *p* < 0.001; ouabain vs. ouabain plus mESCs, *p* < 0.01; Figure 5(b)). These results indicated that reduced auditory connection (caused by both insults) to the central nervous system is improved in mice with mESC transplantation in the chronic neural hearing loss model, possibly reinforcing the use of cell replacement therapies for cochlear neural health and further connection to the brain.

## 5. Discussion

### 5.1. Result Summary

Expecting a positive effect on secondary auditory nerve degeneration in the case of chronic sensorineural hearing impairment with a relatively long prevalence is difficult using cochlear implantation. Therefore, in the present study, mESCs were transplanted directly into the scala tympani, which is mainly composed of neural structures in Rosenthal's canal, as a method to repair or restore damaged nerves. Subsequently, whether the transplanted cells in the cochlea can survive or improve hearing function was investigated. Our established auditory neural hearing loss mouse models, induced acutely by ouabain and chronically by KM, showed increased ABR thresholds and decreased SGN density compared with controls. However, difference was observed in the morphology of the organ of Corti in peripheral auditory organs between the two models. Conversely, in the central auditory pathway, mice in the two models showed the same pattern, a decreased VGLUT1 expression in the CN. Improvement in hearing after mESC transplantation was not observed; however, the survival of transplanted cells in the cochlea was confirmed based on GFP-expressing cells in the cochlea and increased VGLUT1 expression in the CN. The mESC-transplanted mice in both models showed the cells probably supporting cells or perhaps transplanted mESC in the organ of Corti without flat epithelium and no decrease in SGN density. In particular, the distribution and morphology of GFP expression in cochlea were different in the two models. Although GFP-tagged mESCs were observed in all turns of cochlea and Rosenthal's canal in both neural hearing loss models, KM-treated mice in the chronic model formed cellular aggregates and showed weak GFP expression compared with ouabain-treated mice in the acute model. This result indicated that the transplanted mESCs can survive in the perilymphatic region and Rosenthal's canal and their potential to differentiate in a chronic auditory sensorineural hearing loss model.

### 5.2. Difference of Secondary Degeneration and Acute Degeneration of Auditory Neurons

In the present study, a neuropathy model was made by delivering ototoxic reagents (ouabain and KM) into the cochlea via the round window. In a prior report, ouabain-induced neuropathy showed normal levels of DPOAEs and EP, indicating the normal function of outer hair cells and stria vascularis, and selectively induced SGN loss [16]. Although this was not confirmed in the acute group of the current study, structure of the organ of Corti and possibly the structure of stria vascularis remained with probable survival of supporting cells which maintains the endolymphatic conditions. Conversely, KM induced SGN loss and flat epithelium, causing secondary degeneration [19]. In the present study, similar results were obtained in chronic model partially. There was animal showing disruption of the organ of Corti morphology which limits the maintenance of endolymphatic condition. Therefore, the condition within the endolymph was possibly changed due to KM treatment. This difference appears to have affected the cell survival rate and distribution after mESC transplantation. According to present study, the presence of these mESCs apparently resulted in the increase of neural structures in the Rosenthal's canal as shown in Table 1.

Two methods can be suggested for the mechanism of repairing damaged cells or tissues through stem cell therapy. One is that stem cells directly engraft and differentiate into required cell types. The other is that the transplanted donor cells produce a paracrine effect, which stimulates the recipient cells to restore the damaged tissue on their own. Studies of the paracrine effect show that mammalian tissues have reproducible cells and only need to accept appropriate signals to initiate regeneration and repair. In addition, donor-transplanted cells have a long-term effect on tissue regeneration, even though they are short lived. In other words, the presence of the transplanted stem cells is important to initiate tissue repair, but once the patient's own cells are activated, the transplanted stem cells are no longer needed.

A previous study has been reported using umbilical cord blood cells to treat spinal cord injury. The results of this study showed that the transplanted stem cells only existed for 7 to 10 days, but compared to injured animals that did not receive cord blood cells, wound lesions were reduced and the mobility of spinal cord-injured mice was significantly improved [20].

### 5.3. Possible Reasons for Increased Signal Transduction (Maintenance of Neural Structure) in CN

Reportedly, in the acute model, transplanted mESCs are possibly differentiating to the neural lineage without any additional treatment, even outside Rosenthal's canal. The target lineage includes glial cells and astrocytes which are considered supporting cells for maintenance of neural structures in the brain [14]. The differentiation of supportive neural cells or neurons could be the reason for increased neural activities in the CN. Observation of mESCs in Rosenthal's canal in both acute and chronic models might support this theory. Alternatively, paracrine effects of stem cells could be the reason. Secreted trophic factors from stem cells have abilities such as increasing resistance to disease, migration, differentiation, and survival [21]. The trophic factors, which are thought to possibly increase after stem cell transplantation, include interleukins and several neurotrophic factors such as NGF, BDNF, and GDNF [22, 23]. To identify clearly the association between transplanted ESCs and increased neural signaling in the CN, assessing the levels of these trophic factors is necessary. We hypothesize that the combination of the two possible reasons (differentiation and paracrine effect) would lead to the current outcome.

### 5.4. Clinical Implications and Limitations (Future Study Plans)

The present study results indicate the possibility of clinical application of stem cell therapy for hearing rehabilitation. However, regeneration or recovery of damaged neural structures in mouse cochleae was not observed. Considering the short duration of mESC transplantation (2 weeks) and use of pluripotent stem cells, which should be differentiated in multiple steps, the study period was insufficient to observe the transdifferentiation of stem cells to compensate for complex sound transduction systems. Nevertheless, the delivery of stem cells with low efficiency and increase of central signal transduction, possibly through the paracrine effect of pluripotent cells, could be a meaningful result. To substantiate these results further, additional studies are necessary. First, the trophic factors responsible for the paracrine effect should be identified. Second, molecular characteristics of the transplanted cells in different parts of the cochlea should be further analyzed. The additional experiments and confirmation of safe stem cell delivery (allogenic) could support regeneration of neural structures to improve hearing rehabilitation in the near future.

## 6. Conclusion

The survival of transplanted cells in the cochlea was confirmed based on GFP-expressing cells in the cochlea and increased VGLUT1 expression in the CN. In particular, the distribution and morphology of GFP expression in the cochlea was different in the two models. Although GFP-tagged mESCs were observed in all turns of cochlea and Rosenthal's canal in both neural hearing loss models, KM-treated mice in the chronic model formed cellular aggregates and showed weak GFP expression nearby neural structures compared with ouabain-treated mice in the acute model. Improvement in hearing after mESC transplantation was not observed; however, the survival of transplanted cells in the cochlea was confirmed based on GFP-expressing cells in the cochlea and increased VGLUT1 expression in the CN. This result raises the possibility of using stem cell replacement therapy for hearing rehabilitation in patients with chronic nerve damage.

## Figures and Tables

**Table 1:** Status of organ of Corti and Rosenthal's canal in each animal after experiment.

Group	Animal no.	Organ of Corti	Rosenthal's canal
KM only	#3K	Supporting cell remains	Total SGN loss
	#4K	Supporting cell remains	Total SGN loss
	#5K	Flat epithelium	Total SGN loss
	#16K	Supporting cell remains	Intact SGN density
	#17K	Supporting cell remains	Intact SGN density
Ouabain only	#2O	Supporting cell remains	Intact SGN density
	#3O	Supporting cell remains	Partial SGN loss
	#12O	Supporting cell remains	Partial SGN loss
KM + SC	#2KS	Supporting cell remains	Partial SGN loss
	#3 KS	Supporting cell remains	Partial SGN loss
	#9KS	Supporting cell remains	Intact SGN density
	#11KS	Supporting cell remains	Intact SGN density
Ouabain + SC	#17OS	Supporting cell remains	Intact SGN density
	#20OS	Supporting cell remains	Partial SGN loss

KM: kanamycin; SC: stem cell; HC: hair cell; SGN: spiral ganglion neuron.

**Table 2:** Distribution of GFP-positive cells and GFP-positive aggregates in the cochlea after mESC implantation.

Group	Turn	Scala vestibuli	Scala media				Scala tympani
			OC	SGN	SV	Etc.	
Kanamycin + SC	Apical	(++)			(+)		
	Middle	(++)			(+)	(++)	(++)
	Basal	(++)		(+)	(+)		(+)
Ouabain + SC	Apical		(+)				
	Middle						
	Basal		(+)	(+)		(+)	

++: GFP-positive aggregates; +: GFP-positive cells; OC: organ of Corti; SGN: spiral ganglion; SV: stria vascularis.

## Data Availability

The data used to support the findings of this study are available from the corresponding author upon request.

## Additional Points

The English in this document has been checked by at least two professional editors, both native speakers of English. For a certificate, please see: http://www.textcheck.com/certificate/Ek1snR
